# Benchmark Ab Initio Determination of the Conformers, Proton Affinities, and Gas-Phase Basicities of Cysteine

**DOI:** 10.1021/acs.jpca.2c07035

**Published:** 2022-12-16

**Authors:** András B. Nacsa, Gábor Czakó

**Affiliations:** MTA-SZTE Lendület Computational Reaction Dynamics Research Group, Interdisciplinary Excellence Centre and Department of Physical Chemistry and Materials Science, Institute of Chemistry, University of Szeged, Rerrich Béla tér 1, Szeged H-6720, Hungary

## Abstract

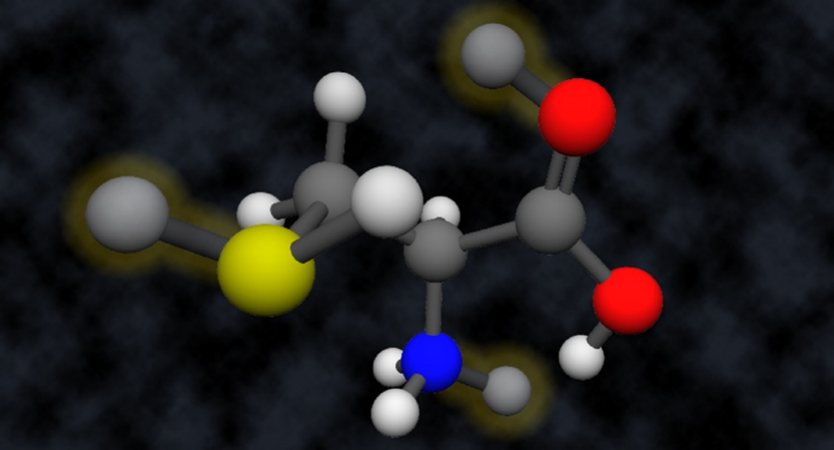

A systematic conformational mapping combined with literature data leads to 85 stable neutral cysteine conformers. The implementation of the same mapping process for the protonated counterparts reveals 21 N-(amino-), 64 O-(carbonyl-), and 37 S-(thiol-)protonated cysteine conformers. Their relative energies and harmonic vibrational frequencies are given at the MP2/aug-cc-pVDZ level of theory. Further benchmark ab initio computations are performed for the 10 lowest-lying neutral and protonated amino acid conformers (for each type) such as CCSD(T)-F12a/cc-pVDZ-F12 geometry optimizations (and frequency computations for cysteine) as well as auxiliary correction computations of the basis set effects up to CCSD(T)-F12b/cc-pVQZ-F12, electron correlation effects up to CCSDT(Q), core correlation effects, second-order Douglass–Kroll relativistic effects, and zero-point energy contributions. Boltzmann-averaged 0 (298.15) K proton affinity and [298.15 K gas-phase basicity] values of cysteine are predicted to be 214.96 (216.39) [208.21], 201.83 (203.55) [194.16], and 193.31 (194.74) [186.40] kcal/mol for N-, O-, and S-protonation, respectively, also considering the previously described auxiliary corrections.

## 1 Introduction

The studies of gas-phase amino acids date back long time ago, both theoretically and experimentally. Their geometries and vibrational frequencies can be used to identify them spectroscopically,^1,2^ especially in interstellar studies,^3,4^ while the proton affinities (PAs) and the gas-phase basicities (GBs) can be helpful to study protonation and formation processes.^5−8^ PAs and GBs are also used in mass spectrometry processes, since these properties govern fragment formation.^9,10^ However, PAs and GBs can also be useful in biochemical applications, since in the knowledge of the solvation energies one can convert these values to liquid state.^11^ The smallest amino acid, glycine, underwent many theoretical and experimental studies;^12−21^ however, if we move forward to more complex molecules, like the cysteine, the number of publications (and in computations, the applied level of theory) drop(s) rapidly due to the more complicated structures (and the higher number of electrons), which statement holds both for the conformers and for the PA/GB values.^1,2,30,31,22−29^ In 2007, a study by Wilke et al.^26^ mapped the complete conformational space at the HF/3-21G level of theory and reoptimized the structures at the MP2/cc-pVTZ level, while the 10 lowest-energy conformers were further optimized at MP2/aug-cc-pV(T+d)Z. In 2013, Freeman^30^ took six conformers with the deepest energy, and they submitted them to geometry optimization and frequency calculations with density functional theory (DFT) and at the MP2/6-311+G(d,p) level of theory. Furthermore, they performed CCSD(T) and QCISD(T) single-point energy calculations with the cc-pVTZ basis set. There are recent studies^32−35^ using algorithms and machine learning to find conformers, but the finally applied methods are either DFT or MP2. These techniques are based on the exploration of the PES of the target molecule going further than stochastical (Monte Carlo sampling) or deterministic (molecular dynamics) approaches by metaheuristic (e.g., nature inspired algorithms) methods to provide cost-efficient alternatives. For PA and GB, there are a few theoretical studies, the most recent ones are from 2011,^27,29^ where the computational level is either based on MP2 with single-point coupled-cluster computations^27^ or DFT.^29^ Another weakness present in other studies that they only consider the lowest-energy conformers for the protonation and/or only one protonation site.

After our high-level theoretical studies on glycine and alanine,^16,36^ here we present an explicitly correlated ab initio investigation on the conformers of the cysteine and its protonated counterparts as well as PA and GB values. From the qualitative side, we map the extended conformational space of the neutral and protonated amino acid; in the latter case, we consider three possible protonation sites. These investigations hopefully reveal new conformers in addition to previous studies for both forms of cysteine. To achieve quantitatively benchmark data, in the case of the most important structures, we provide CCSD(T)-F12a/cc-pVDZ-F12 geometries, which is a unique feature of our work, and harmonic vibrational frequencies (in the case of protonated amino acid, only MP2/aug-cc-pVDZ frequencies), while we consider basis effects up to the cc-pVQZ-F12 basis set. Many other auxiliary corrections are also included in this work: post-(T) corrections up to CCSDT(Q), core–core, core–valence correlation, and finally the scalar relativity for cysteine and protonated cysteine molecules. These are necessary to acquire high accuracy, based on our previous study.^16^ Finally, we determine PA and GB values based on these new ab initio results and on statistical mechanics, these values can be used in mass spectroscopy measurements and in protonation processes, as, e.g., the fragmentation for particular amide bonds in peptides can be related to the PA values of the individual amino acids.^9,10^

## 2 Computational Details

### 2.1 Conformers of Cysteine and Protonated Cysteine

The first objective is to find as many conformers of cysteine and its protonated counterparts as possible. Our investigation of the neutral amino acid is based on the global minimum structure of Wilke et al.^26^ To describe the conformational space, we rotate the parts of this conformer responsible for the main internal rotations, namely, the amino, carboxyl, hydroxyl, and thiol groups and the sidechain by 60°. This means six steps for each groups (0° and 360° are the same structure) resulting in 6^5^ = 7776 initial geometries, and we cannot decrease this number since the symmetry of the system is *C*_1_. Initially for the geometry optimization we use the MP2 method with different basis sets: 3-21G,^37^ 6-31G,^37^ 6-31++G,^37^ 6-31G**,^37^ 6-31++G**,^37^ and cc-pVDZ.^38^ In addition to this, we also utilize the MP2/cc-pVTZ^38,39^ and HF/3-21G^37^ levels of theory, and for the latter, we increase the resolution of the internal rotation to 30° in the case of the amino group and the side chain.

The next step is to gather information about the protonated cysteine. Based on chemical intuition, we have four protonation sites: the hydroxyl, carbonyl, amino, and thiol groups, leading to ^+^(H_2_O)OC–CH–(NH_2_)–CH_2_–SH, ^+^(HO)_2_C–CH–(NH_2_)–CH_2_–SH, HOOC–CH–(NH_3_^+^)–CH_2_–SH, and HOOC–CH–(NH_2_)–CH_2_–SH_2_^+^, respectively. To validate these assumptions, we take the previously found cysteine global minimum and attach a proton to the corresponding groups in two variations. For the hydroxyl and thiol groups, the difference between their two cases is that H–O–H and H–S–H planes are perpendicular; in the case of the amino group, it is rotated with 60° while the two carbonyl-protonated conformers differ in the *cis*–*trans* alignment. For the verification, we use the MP2/aug-cc-pVDZ level of theory. We indicate ahead, that similarly to glycine,^16^ we find that the protonation of the hydroxyl group does not lead to a stable conformer, whereas the thiol group on the sidechain can be protonated along with the carbonyl and amino ones.

To map the protonated conformers, we use a similar procedure as previously described for cysteine, but here there are some symmetry properties that are worth exploiting during the systematic search. For the N-(amino-)protonated case, the protonated amino group has *C*_3v_ symmetry, reducing the number of initial computations to 6^4^ × 2 = 2592. The O-(carbonyl) protonation introduces a new hydroxyl group that we need to rotate and this would lead to 6^6^ = 46,656 initial geometries, but recognizing the *C*_2v_ symmetry of the {C(OH)_2_} group, it can be halved to 23,328. The protonation of the thiol group (S-protonation) does not make new rotations necessary; however, we cannot profit from symmetry, so we have 6^5^ = 7776 initial configurations. We note in advance that based on conformer search for the neutral cysteine, the use of the MP2 method with the 6-31++G** and cc-pVDZ basis sets is advised for mapping the conformational potential of the protonated species.

To distinguish the optimized results, we use the same strategy in both cases and later on too. First, we sort the structures by their energies relative to the minimum and by their *A* rotational constant. We declare two geometries different if there is a minimum difference of 0.01 kcal/mol in the relative energy and/or >3 × 10^–4^ cm^–1^ deviation in *A*; these limits are based on the analysis of the results. Until this point, we do not differentiate transition states and minima, but to do this, we further optimize the conformers and compute the harmonic frequencies using the MP2 method with the correlation-consistent aug-cc-pVDZ basis set.^38^ We use Molpro^40^ and MRCC^41,42^ programs to perform the ab initio computations and our python code for data analysis.

### 2.2 Benchmark Structures and Energies

We subject the 10 lowest-lying minima of the neutral and protonated amino acid to higher level computations with the explicitly correlated coupled-cluster singles, doubles and perturbative triples method^43,44^ (CCSD(T)-F12a) with cc-pVDZ-F12^45^ (geometry and frequencies (the latter is only for neutral Cys)). Using the cc-pVTZ-F12 and cc-pVQZ-F12 basis sets, we perform single-point energy computations. In the case of the cc-pVQZ-F12 basis set, we use both the CCSD(T)-F12a and CCSD(T)-F12b methods, and the latter is utilized for the final benchmark energy computations as F12b is expected to be slightly more accurate than F12a with the large QZ basis set.^40^ In addition, we compute the following energy corrections based on the CCSD(T)-F12a/cc-pVDZ-F12 geometries:

The coupled-cluster triples (δT)^46^ and perturbative quadruples (δ(Q))^47^ corrections are determined with the 6-31G basis set. Previous investigations^16^ compared to smaller (3-21G) and larger (cc-pVDZ) basis sets showed that the 6-31G basis set is sufficient to provide satisfying accuracy for glycine.
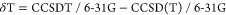
1
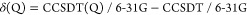
2

We compute all-electron (AE) and frozen-core (FC) energies at the CCSD(T)-F12a/cc-pCVTZ-F12 level of theory^48^ and define the core correlation correction as
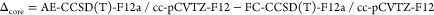
3

FC methods only correlate the electrons on the valence shells, whereas utilizing AE computations, we correlate the 1s^2^ electrons for the C, N, and O atoms and the 2s^2^, 2p^6^ electrons for the S atom.

To determine the scalar relativistic effects with computing the second-order Douglas–Kroll^49^ (DK) relativistic energies, we use the AE-CCSD(T)^50^ method with the aug-cc-pwCVTZ-DK^51^ basis set:
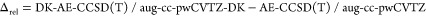
4

Zero-point energy corrections (Δ_ZPE_) are based on the MP2/aug-cc-pVDZ harmonic frequency results.

At the end, we obtain the benchmark electronic (equilibrium) and adiabatic (ZPE corrected) energies by summarizing the cc-pVQZ-F12 single-point energies with the corrections:
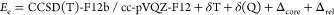
5
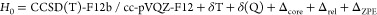
6

### 2.3 Proton Affinity and Gas-Phase Basicity Computations

The PA and the GB equal the enthalpy (Δ*H*) and the Gibbs free energy (Δ*G*) change of the following gaseous reaction:
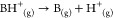
R1

BH^+^ is the protonated conjugate acid, B is the analogue gaseous base, and H^+^ is a free proton. We employ the rigid rotor and harmonic oscillator models in addition to our ab initio computations to determine the PA and GB values. Temperature corrections can be obtained for the translational, vibrational, and rotational enthalpies and entropies applying standard statistical mechanics expressions. Since we have conformer mixtures, we need to calculate the population of each one. We perform this using a simple Boltzmann-distribution:
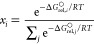
7where *x_i_* is the relative population of the *i*th conformer, and Δ*G*_rel, *i*_^○^ is the molar standard Gibbs free energy of the *i*th conformer relative to the most stable conformer.

## 3 Results and Discussion

### 3.1 Conformers of the Neutral and the Protonated Cysteine

The summary of the systematic conformer search for cysteine can be seen in Table 1 based on the MP2 method with six different basis sets. The internal rotations were mapped by 60° in the corresponding internal torsional angles for every basis set. These initial geometries were optimized, and if there was convergence, we assigned them to a conformer. Finally, we obtain 48, 44, 39, 51, 49, and 50 stationary points and 45, 41, 37, 47, 45, and 43 minima from the 3-21G, 6-31G, 6-31++G, 6-31G**, cc-pVDZ, and 6-31++G** basis sets, respectively, at the MP2/aug-cc-pVDZ level of theory. We distinguish unique stationary points and minima, which were not described by smaller basis sets. All in all, we got 55 different minimum conformers with these methods. The 6-31++G underperforms both the larger and the smaller basis sets; however, we cannot obtain general information about the usefulness of the diffuse functions since while adding them to the 6-31G** basis set decreases the number of minima from 47 to 43, five unique conformers get located this way. For larger systems or when the goal is not to find all the conformers, 3-21G or 6-31G** can be handy, since their computation cost is quite cheap, while they lead to a high number of conformers (the former is better in the <2.5 kcal/mol region, it provided all of the ones found in present work under this energy limit). For smaller molecules, where it is feasible, it seems that it is worth using even larger basis sets than operated here, but not as a standalone, but as expansions to smaller ones.

**Table 1:** Number of Stationary Points and Minima Found While Mapping the Conformational Space of the Neutral Cysteine Using the MP2 Method with Different Basis Sets

	3-21G	6-31G	6-31++G	6-31G**	cc-pVDZ	6-31++G**
stationary points	48	44	39	51	49	50
unique^a^ stationary points		3	0	7	5	11
minima	45	41	37	47	45	43
unique^a^ minima		2	0	5	2	5

a By unique, we mean a geometry that was not found with smaller basis set(s).

**Figure 1:**
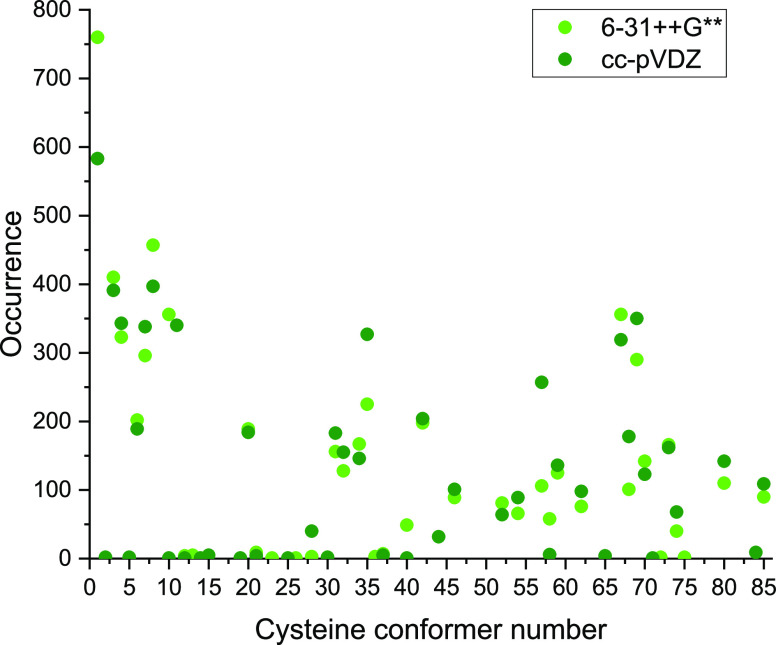
Occurrence of the different cysteine conformers during the mapping of the conformational space with the 6-31++G** and cc-pVDZ basis sets using the MP2 method.

Nonetheless, we were not satisfied as we compared our results with the literature. The neutral cysteine has been studied both in the past^22,24,26,29,52,53^ and recently,^32,33,35^ and there is no absolute agreement in the number of cysteine minima. The most comprehensive study was done by Wilke et al.,^26^ and they found 71 minimum conformers at the MP2/cc-pVTZ level. To directly compare our results, we reoptimized our structures with the former level of theory and performed vibrational frequency computations to check if the difference origins from imaginary frequencies, but that was not the case. The number of missing conformers was not just simply 71–55 = 16, but 31, since some of our results were not present in their work. It is important to note, that at this level of theory, we had two new conformers with less than 3 kcal/mol relative energy to the global minimum. After this, we tried thinking backward: we reoptimized the missing minima and computed their frequencies at the MP2/aug-cc-pVDZ level. We could reduce the 31 by one, since two geometries converged to the same structure and the frequencies showed that these are also indeed minima. One difference in the present work is the starting level of theory: we tried the reproduction with HF/3-21G used by ref (26). This way we found 2 of the missing 30, so 28 more were needed. Also, it is worth noting that the rotation with HF/3-21G also resulted in some of the conformers that was not present in the previous work. Another difference was in the resolution of the rotation: previous work did not rotate with 60° uniformly, but with 30° for the amino group and the sidechain and 120° for the other torsional angles. We also tried rotating the two former parts of the cysteine by not 60° but with 30° systematically at the HF/3-21G level. This produced us only one more of the missing conformers. Finally, we adapted the remaining 27 conformers to our 55 + 2 + 1, meaning we have 85 conformers at the MP2/aug-cc-pVDZ level. We cannot come to conclusion what causes the difference, we also tried to analyze the structure of the missing conformers, but this did not show any useful information regarding any solution or explanation. The inequality might rise from using different electronic structure packages (different optimization method) or a different initial geometry, which gives the base of this kind of conformer search. The occurrence of each conformer during the conformational mapping with MP2/6-31++G** and MP2/cc-pVDZ methods can be seen in Figure 1. In general, if one geometry is more or less likely to be found with one basis set, it is the same with the other, but there are a few exceptions. For example, conformer number 10 was found in 356 cases with the former basis set, while only once with the latter. The geometries which were not located by both of the basis sets are not frequent as they are found in less than 10 times in every case, usually 1 or 2 times. The two exceptions are conformer number 11 and 44, which present 340 and 32 times in the cc-pVDZ data set, respectively. 6-31++G** found 6, while cc-pVDZ located 10 conformers, which were not identified by the other basis set. As many structures are not so frequent, this finding also indicates that this kind of conformational space mapping can be very sensitive to the initial geometry, leading to the conclusion that there might be some conformers neglected even in the not so high-energy region.

**Figure 2:**
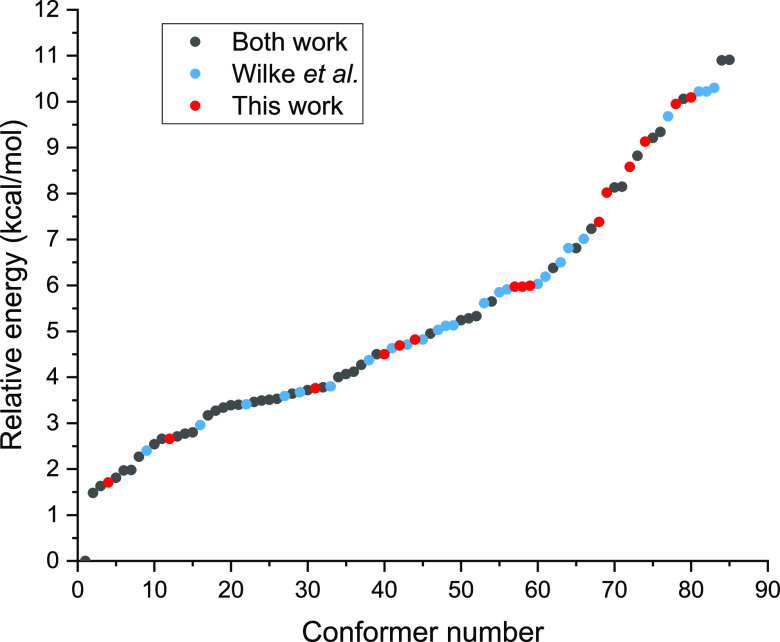
MP2/aug-cc-pVDZ relative energies of the 85 cysteine conformers discussed in present work. Red means that the given conformer was only located by this work, blue means it was only found by a previous study^26^ while gray means it was identified by both studies.

The relative energy distribution of the aforementioned conformers can be seen in Figure 2, where we noted the origin of the structures. The highest energy is 10.91 kcal/mol, while the lowest is 1.48 kcal/mol, relative to the global minimum, at the MP2/aug-cc-pVDZ level of theory. Between these two limits, the values are fairly continuous, with three bigger gaps, one at number 68 (0.64 kcal/mol), the middle one at number 71 (0.43 kcal/mol), and the last one at number 83 (0.60 kcal/mol). We also included the origin, i.e., ref (26) or this work, of each conformer in the figure. It seems that the two studies are nice complementary to each other, since there are conformers found by only one of them in practically every energy region.

**Figure 3:**
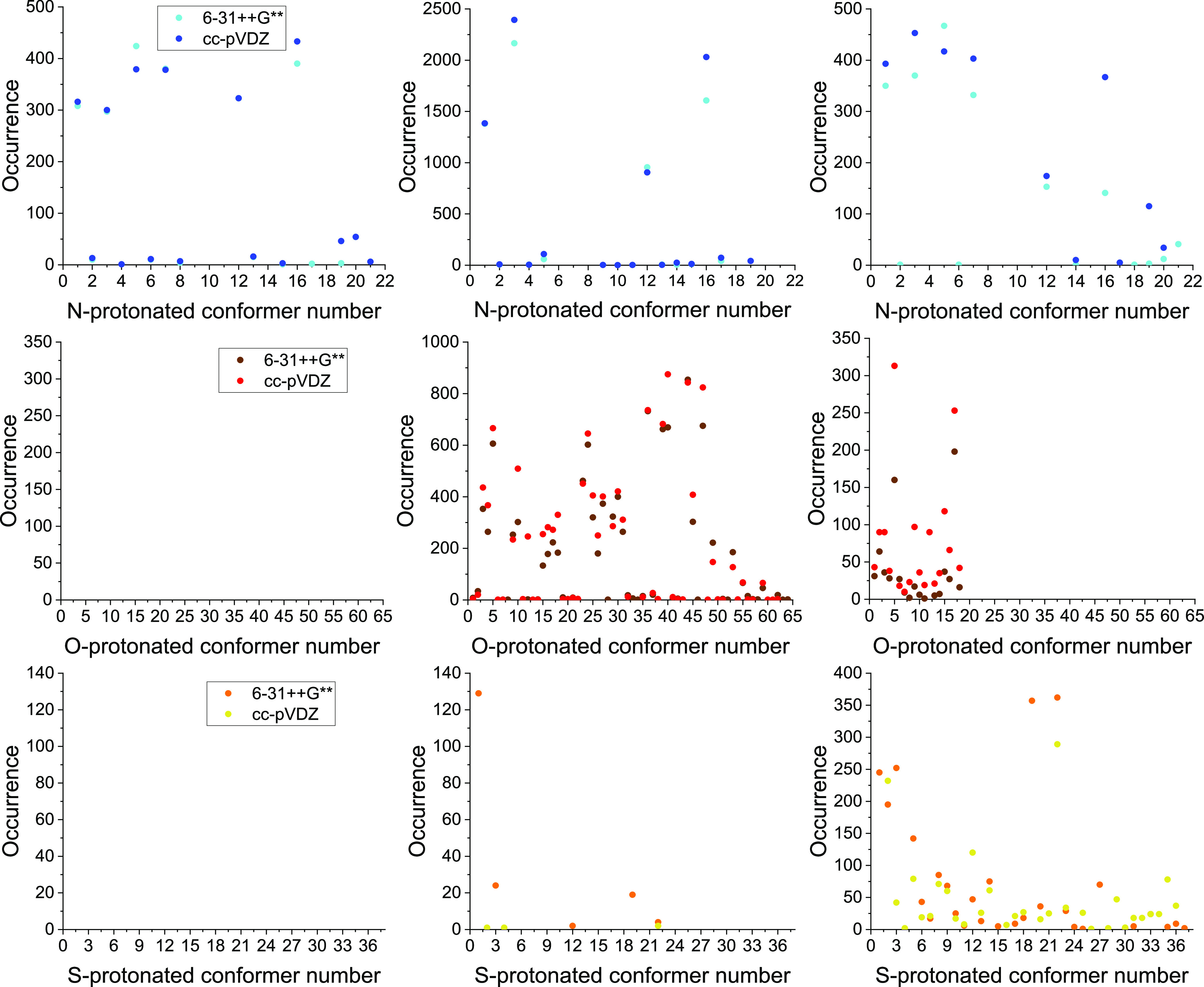
Occurrence of the different N-, O-, and S-protonated cysteine conformers (first, second, and third rows, respectively) during the mapping of the conformational space with the 6-31++G** and cc-pVDZ basis sets using the MP2 method. The three columns represent the three different mapping process, based on the rotation of an amino- (left), carbonyl- (middle), or thiol- (right) protonated initial geometry.

**Figure 4:**
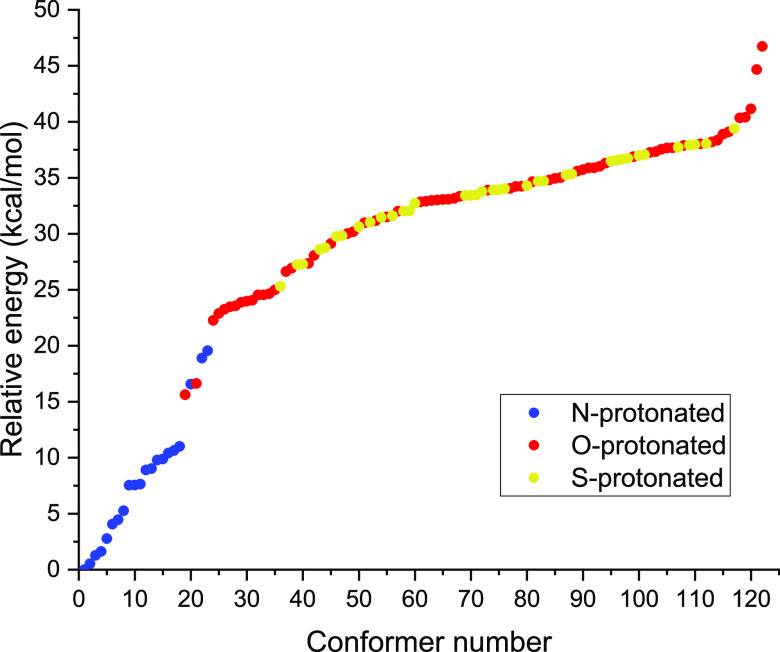
MP2/aug-cc-pVDZ relative energies of the 122 protonated cysteine conformers. There are 21 N- (blue), 64 O- (red), and 37 S-protonated (yellow) structures.

Protonation tests showed similar results to that in the case of glycine and alanine: the cysteine protonation can happen at every predicted site except the hydroxyl, so we can categorize our structures as N-(amino-), O-(carbonyl-), and S-(thiol-)protonated conformers. In the case of the protonated cysteine, we choose the cc-pVDZ and 6-31++G** basis sets with the MP2 method for the mapping based on the results obtained for the neutral cysteine. These two combined provided all of the cysteine conformers found with our original method, yet they still had structures that were found by only one of them. The final conformers were optimized at the MP2/aug-cc-pVDZ level of theory along with harmonic vibrational frequency computations, and the results were 21, 64, and 37 N-, O-, and S-protonated conformers, respectively, along with some “byproduct” structures. While the global minimum agrees in our study with previous reports,^29,53^ the number of protonated conformers has been greatly increased as compared to 21 in ref (29) or 6 in ref (53). We can explain the relatively high number of the O-protonated conformers by considering the conformational space: in that case we have a new important torsional angle which leads to more stable conformers just like in the case of glycine and alanine.^16,36^ The occurrence of the conformers upon the three mapping can be seen in Figure 3. With the initially N-protonated data set (first column), we only got N-protonated structures, 16 of the final 21. The two basis sets are in good agreement with the number of points leading to the same conformer, and it can be seen that the 6-31++G** found 1 (N17), while the cc-pVDZ found 2 (N4 and N21) minima, that were not present in the other data set with occurrences of 2, 1, and 6, respectively. Many of the originally O-protonated initial structures (second column) converged to N- and S-protonated ones, the former ones are much deeper in energy, while the latter ones mix with the carbonyl-protonated structures in energy (the energy distribution will be discussed later). The amino-protonated N9, N10, and N11 conformers were located only by this mapping process with the cc-pVDZ basis set from 2, 1, and 2 initial structures (which already seem very small portion, especially if we keep in mind, that in this case we have more than 20,000 points in comparison to the over 2000 initially amino-protonated structures). We find 7 of the 37 thiol-protonated minima, but none of them are exclusive to this data set. Finally, we find all the final 64 carbonyl-protonated structures. Again the number of initial geometries leading to one minimum is usually in good agreement between the two basis sets, and the minima limited to one of them (9 for 6-31++G** and 8 for cc-pVDZ) are not frequent. The initially thiol-protonated data set (third column) leads to 37 of the 37 S-protonated geometries, also to many amino- (14 of 21) and some carbonyl- (the first 18 of the 64) protonated conformers for the same energetic reasons. Here, we can observe much difference between the two basis sets with respect to the previous searches, and the occurrence is not in a good agreement between them. For example, the lowest conformer, S1 was only located by 6-31++G**, but quite frequently, 245 times (6 unique minima for 6-31++G** and 8 for cc-pVDZ)! From these mapping processes, we can draw the following conclusions: these two basis sets work well with each other as it was found in the case of the neutral cysteine. The rarity of some minima suggests that this kind of search is very sensitive to the initial geometry, and there might be other, yet neglected structures even in the low energy region. The energy distribution of these minima can be seen in Figure 4. The deepest energy belongs to the amino-protonated structures, while comparing the other sites, the carbonyl-protonated minimum has ∼15.6 kcal/mol while the thiol-protonated minimum has ∼25.3 kcal/mol relative energy with respect to the N-protonated minimum and as one can see, the protonation of the different sites “overlap,” as the highest N-protonated conformer has higher relative energy than the lowest two O-protonated ones. Also, from the relative energy levels of the S-protonation the carbonyl- and thiol-protonated structures mix. This also explains that during the systematic rotations many initial structures converged into a geometry with a different protonation site.

### 3.2 Byproduct Conformers

Upon searching for the minimum geometries, we found some “byproduct” structures that are not relevant for our work, but they are worth mentioning. MP2/aug-cc-pVDZ harmonic frequency calculations proved that all of them are indeed minima. The relative energies are given with respect to the lowest-energy cysteine conformer for the structures found while mapping the neutral cysteine, and to the lowest-energy N-protonated cysteine for the structures found during the protonated cysteine conformer search.

**Figure 5:**
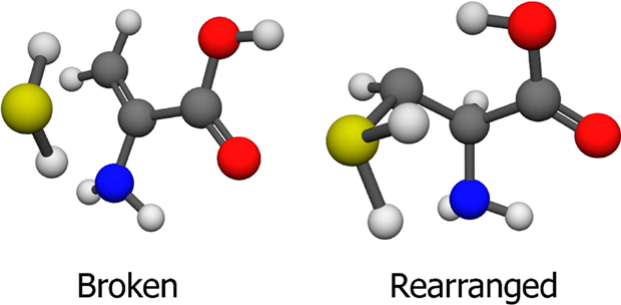
Two typical byproduct minima of the neutral cysteine conformer search at the MP2/aug-cc-pVDZ level of theory. There are 23 Broken and 20 Rearranged structures.

For the neutral amino acid, we could categorize them into two classes, called “Broken” and “Rearranged” as can be seen in Figure 5. For the former ones, one can observe two bond breaks: an S–C and an H–C, the hydrogen joins to the thiol group and the SH_2_ positions itself away from the main molecule. The 23 geometries lie in the 14.86–23.67 kcal/mol relative energy range. Our last cysteine minimum has a relative energy of 10.91 kcal/mol, so there is a gap in-between, but this indicates that one should take extra caution in the event of conformer search as in bigger molecules the difference might disappear. We suggest that it is useful to have basic visual confirmation, or in the case of large data sets, some kind of restriction on the atomic bond lengths to determine, whether we found the target molecule or something else. The other class of the byproducts is the “Rearranged.” These geometries at the first glance might look like a thiol-protonated conformer, but the origin of the proton is the carbon atom of the side chain. The 20 conformers exist in the 65.91–76.87 kcal/mol relative energy regime, so these are not so easily mistaken as a neutral cysteine. In agreement with our previous suggestion, these minima can be filtered by the constraints.

**Figure 6:**
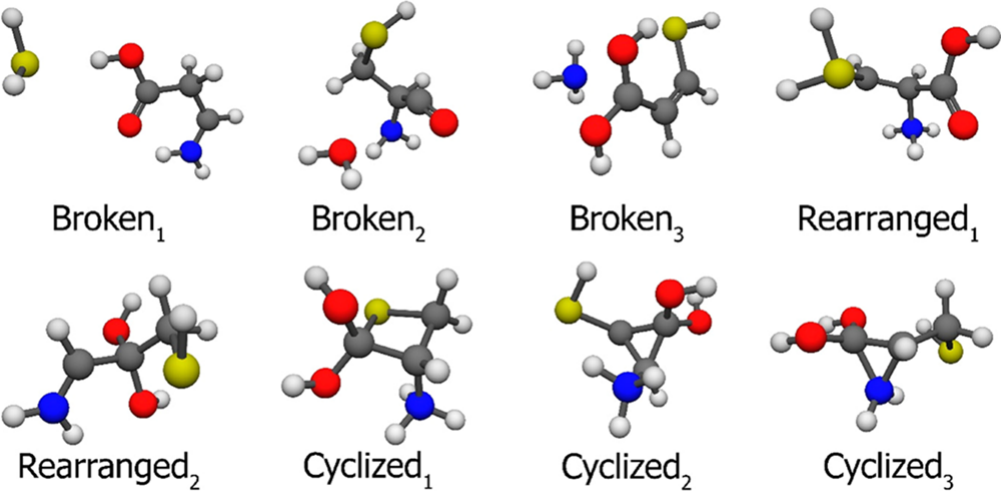
Three typical byproduct minima of the protonated cysteine conformer search at the MP2/aug-cc-pVDZ level of theory. There are 40 + 5 + 2(47) Broken, 6 + 1 Rearranged, and 1 + 15 + 9(25) Cyclized structures.

For the protonated cysteine, we found three classes as it can be seen in Figure 6: “Broken,” “Rearranged”, and “Cyclized,” respectively. We have three types of bond breakages differing whether we find SH_2_ (Broken_1_), OH_2_ (Broken_2_), or NH_3_ (Broken_3_) along with the remnant larger molecule fragment. There are 40, 5, and 2 minima in the 8.00–49.34 kcal/mol and 31.82–41.16 kcal/mol relative energy ranges and with 26.76 and 29.97 kcal/mol relative energies, respectively. The gap between the target molecules (protonated cysteine) and the byproducts ceases to exist in this case, so separation based on the number of bonds and their lengths comes handy. One could argue that the system with the OH_2_ is not broken, rather hydroxyl-protonated, but the elongated C–O bond (∼2.7 Å) tells us that it is some kind of a molecular complex at best, in accordance with our protonation site tests. We have six Rearranged_1_ structures, where a hydrogen atom from the carbon atom of the sidechain changes its position to a possible protonation site. The relative energies are in the 59.45–64.92 kcal/mol region. We found one Rearranged_2_ conformer with a relative energy of 25.37 kcal/mol, where the N- and O-containing ligands switch carbon atoms. There is a new class, called Cyclized since here we observed some kind of ring formation in three different variations. The first one contains a four-atom ring, C–C–C–S, we found one of this type with a relative energy of 20.61 kcal/mol. Cyclized_2_ and Cyclized_3_ structures have three-atom rings, C–C–C and C–C–N. Three structures with their rings made of carbon atoms have 37.03, 37.25, and 37.57 kcal/mol relative energies. The remaining 12 lies in the 62.18–73.61 kcal/mol regime. The nine C–C–N ringed conformers are in the 36.00–39.83 kcal/mol relative energy range. The byproduct structures and their relative energies can be found in the Supporting Information.

### 3.3 Further Benchmark Structures and Energies

Applying the Boltzmann-distribution shows us that the structures with higher energy level quickly become negligible for reaching the desired high accuracy for PA and GB. Therefore, we took the 10 conformers (both the neutral and protonated cysteine) with the lowest relative energies (it is important to note that the order of the conformers was determined at the MP2/aug-cc-pVDZ level of theory, since there are multiple changes in the final lineup with the adjustment of the level of theory) and subjected them under further analysis.

**Figure 7:**
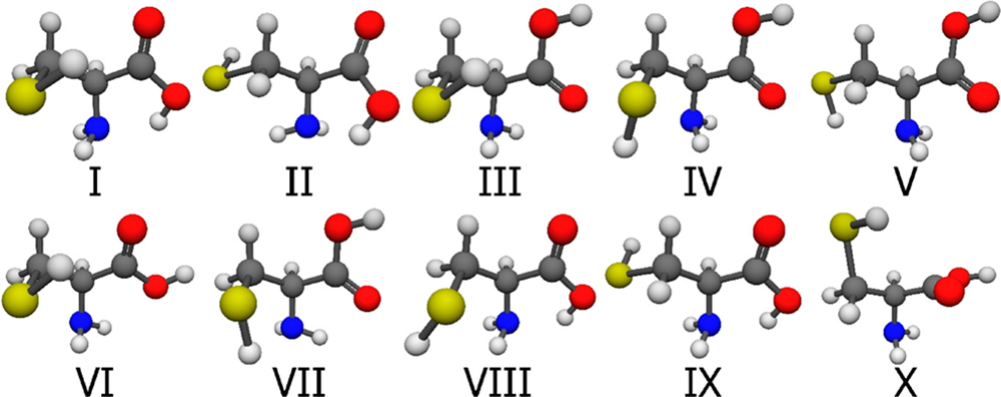
CCSD(T)-F12a/cc-pVDZ-F12 geometries of the first 10 cysteine conformers. It is important to note that the selection was based on the MP2/aug-cc-pVDZ relative energies and the numbering reflects the CCSD(T)-F12b/cc-pVQZ-F12 energy order.

**Figure 8:**
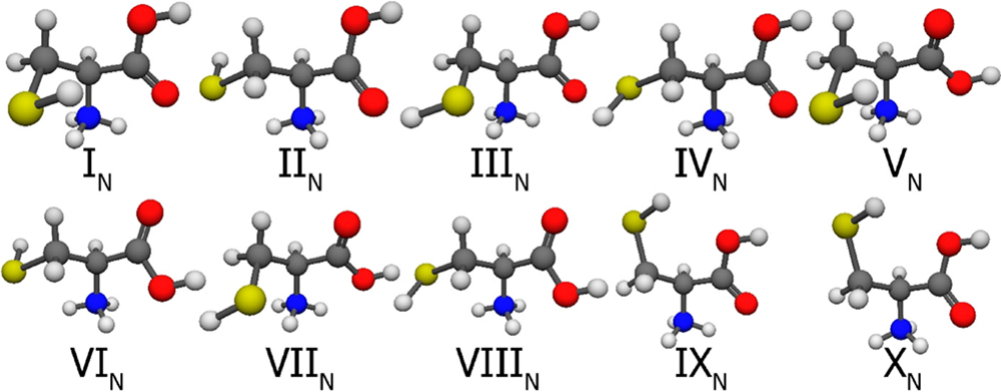
CCSD(T)-F12a/cc-pVDZ-F12 geometries of the first 10 N-protonated cysteine conformers. It is important to note that the selection was based on the MP2/aug-cc-pVDZ relative energies and the numbering reflects the CCSD(T)-F12b/cc-pVQZ-F12 energy order.

**Figure 9:**
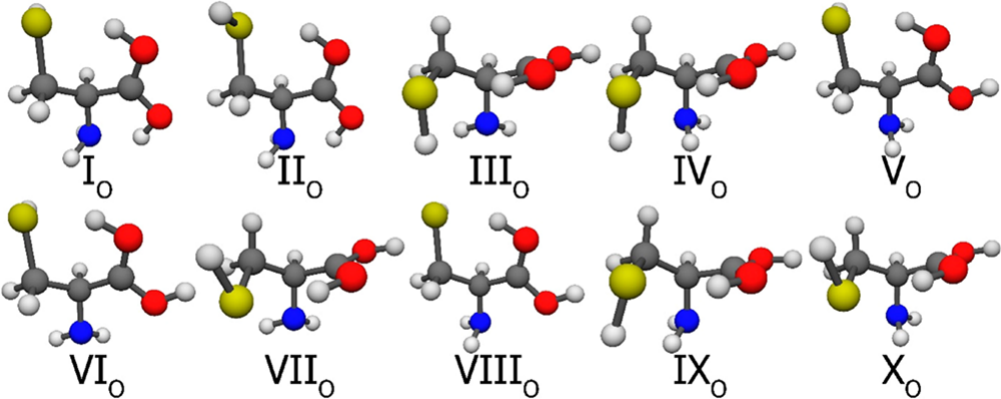
CCSD(T)-F12a/cc-pVDZ-F12 geometries of the first 10 O-protonated cysteine conformers. It is important to note that the selection was based on the MP2/aug-cc-pVDZ relative energies and the numbering reflects the CCSD(T)-F12b/cc-pVQZ-F12 energy order.

**Figure 10:**
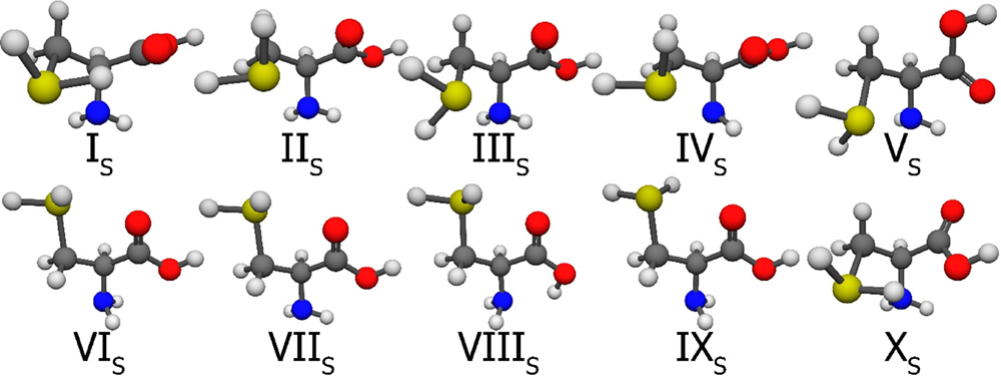
CCSD(T)-F12a/cc-pVDZ-F12 geometries of the first 10 S-protonated cysteine conformers. It is important to note that the selection was based on the MP2/aug-cc-pVDZ relative energies and the numbering reflects the CCSD(T)-F12b/cc-pVQZ-F12 energy.

The 10 selected neutral Cys conformers can be seen in Figure 7, the 10 amino-protonated in Figure 8, the 10 carbonyl-protonated in Figure 9, and finally, the 10 thiol-protonated in Figure 10. The geometries are at the CCSD(T)-F12a/cc-pVDZ-F12 level of theory, the notation of Roman numerals indicates increasing CCSD(T)-F12b/cc-pVQZ-F12 single-point energies while the subscript letter refers to the protonation site. In general, we can say that the structures are usually stabilized by one or more intramolecular hydrogen bond(s), as expected. The N-protonation hinders the acceptor role of the N atom, while the O protonation blocks the same character of the carbonyl O atom. The cysteine global minimum resembles to the II_N_^16^ and II_a_^36^ structures of the glycine and the alanine with respect to the dihedral angles related to the α and β carbon atoms, and it is also stabilized with a hydrogen bond between the lone electron pair of the N atom and the H atom of the hydroxyl group. There is a much weaker interaction between the H atom of the thiol group and the double bonded oxygen, as their distance is ∼2.7 Å. The amino-protonated minimum, along with the glycine and alanine minima, has a strong intramolecular H-bond between the protonated amino group and the lone electron pair of the carbonyl group. In the case of the two smaller amino acids, we cannot observe these kinds of interactions for O protonation, whereas the O-protonated cysteine has stabilizing hydrogen bonds in many of its conformers, in the minimum, it has a strong O–H···N and a weaker O–H···S interaction. The S-protonated minimum is stabilized by the interaction between one of the hydrogen atoms on the protonated thiol group and the N and O atoms.

The relative energies, enthalpies, free energies, and auxiliary energy corrections of the neutral cysteine can be seen in Table 2. The relative energy order changes at many places at higher level of theory, coupled-cluster order is noted by Roman numerals, while the MP2 order by Arabic numerals. The difference between the MP2/aug-cc-pVDZ and CCSD(T)-F12a/cc-pVDZ-F12 relative energies can be separated into two sources: one part originates of course from the higher level of theory, while the other part’s source is the change in geometry. The latter can be calculated as the difference between the CCSD(T)-F12a/cc-pVDZ-F12 energies at the MP2/aug-cc-pVDZ and CCSD(T)-F12a/cc-pVDZ-F12 structures. Here, this contribution is not more than a few times 10^–2^ kcal/mol, so the MP2/aug-cc-pVDZ structure is quite good. It can also be seen that the MP2/aug-cc-pVDZ and CCSD(T)-F12a/cc-pVDZ-F12 relative energies match within a few 10^–2^ kcal/mol, except for the two last conformers, where the changes are −0.37 and +0.15 kcal/mol, respectively. Single-point computations with cc-pVTZ-F12 and cc-pVQZ-F12 basis sets show good agreement between them, correcting the cc-pVDZ-F12 relative energies with 0.01–0.04 kcal/mol, proving the good basis-set convergence. The cc-pVQZ-F12 basis set was utilized both with the CCSD(T)-F12a and CCSD(T)-F12b methods. In general, for cc-pVQZ-F12 basis set, the F12b method is suggested,^40^ in relative energies we do not see much difference, as it only reaches 0.01 kcal/mol for conformer III. From the complementary corrections, correlating the inner electrons seems necessary to achieve high accuracy, as the core correlation value can be as high as 0.05 kcal/mol (for conformer X), and in 0.03 kcal/mol on average. The Douglass–Kroll relativistic effects seem to be less important, as the relativistic correction stays below 0.02 kcal/mol (negative value in every case), but usually it is 0.01 kcal/mol. The post-(T) corrections are important, as the sum of the δT and δ(Q) contributions is 0.05–0.06 kcal/mol for most of the conformers, the exceptions are conformer II with −0.01 kcal/mol value (the only negative one), conformer IX, where it can be neglected, and conformer X, where it is 0.04 kcal/mol. The two components (δT and δ(Q)) are similar in most of the cases. In summary, these improvements change the relative energies by 0.05 kcal/mol, but for some cases, the corrections reach 0.08–0.09 kcal/mol. Conformers VI and VII are very close in energy, with a higher level of theory, conformer VI seems to be deeper in energy, but after adding the corrections, VII exchanges with VI in the relative energy order. For the neutral amino acid conformers, we computed MP2/aug-cc-pVDZ and CCSD(T)-F12a/cc-pVDZ-F12 harmonic vibrational frequencies. Considering the computational cost vs the increase in the accuracy, the latter is not advised for protonated amino acids (as it was found out), but we were interested in the order of magnitude of the difference between the two methods, to estimate the accuracy of our final results. In Table 2, we display the MP2/aug-cc-pVDZ zero-point energy correction; on average, they differ from the CCSD(T)-F12a data by 0.08 kcal/mol. Our computations consider a harmonic oscillator model, and the neglected anharmonicity can have an effect of 0.01–0.1 kcal/mol on the ZPE corrections as shown in previous work for amino acids.^54,55^ After the addition of the ZPE corrections, we finally get the 0 K enthalpy values and we can see changes in the order in the case of conformers II–III, VIII–IX, and the difference between VI–VII enlarges. After computing the thermal contributions with the help of statistical thermodynamics, we get the enthalpy values at 298.15 K. Here, we also have changes, as the final order is I, III, II, IV, VII, V, VI, VIII, X, and IX, and the change in the relative enthalpy of conformers IX and X seems to be an outlier. Subtracting the *TS* term, where *T* and *S* denote the temperature and entropy, respectively, we get the free energy values, and the value of this contribution varies for the conformers. The thermal corrections are very sensitive to low frequency vibrations, as mentioned before in the case of glycine,^16^ so the 298.15 K enthalpy and Gibbs free energy values might not be as accurate as our other results.

**Table 2:** Benchmark Relative Energies, Corrections, Relative Enthalpies (at 0 and 298.15 K), and Relative Gibbs Free Energies (298.15 K) of the 10 Lowest-Lying Cysteine Conformers^a^

Name^b^	MP2	CC//MP2	CCSD(T)-F12a			CCSD(T)-F12b	Δ_core_^i^	Δ_rel._^j^	δT^k^	δ(Q)^l^	Δ*E*_e_^m^	Δ_ZPE_^n^	Δ*H*_0_^o^	Δ*H*_298.15_^p^	Δ*G*_298.15_^q^
	aVDZ^c^	DZ^d^	DZ^e^	TZ^f^	QZ^g^	QZ^h^									
I/1	0.00	0.00	0.00	0.00	0.00	0.00	+0.00	+0.00	+0.00	+0.00	0.00	+0.00	0.00	0.00	0.00
II/2	1.48	1.48	1.49	1.53	1.53	1.53	+0.01	–0.01	+0.00	–0.01	1.52	–0.06	1.46	1.58	1.32
III/3	1.63	1.63	1.61	1.62	1.61	1.60	+0.03	–0.02	+0.02	+0.02	1.66	–0.50	1.16	1.42	0.43
IV/4	1.71	1.75	1.74	1.75	1.74	1.74	+0.04	–0.02	+0.02	+0.03	1.81	–0.44	1.37	1.62	0.72
V/5	1.81	1.78	1.78	1.81	1.80	1.80	+0.04	–0.01	+0.03	+0.02	1.87	–0.44	1.43	1.68	0.81
VI/7	1.98	1.95	1.95	1.95	1.94	1.94	+0.04	–0.01	+0.03	+0.02	2.03	–0.28	1.75	1.86	1.26
VII/6	1.97	1.95	1.95	1.96	1.95	1.95	+0.01	+0.00	+0.03	+0.03	2.02	–0.55	1.46	1.65	1.11
VIII/8	2.27	2.10	2.10	2.11	2.11	2.11	+0.01	–0.02	–0.01	+0.01	2.09	–0.15	1.94	2.08	1.73
IX/10	2.54	2.15	2.17	2.19	2.19	2.19	+0.02	–0.01	+0.00	+0.00	2.20	–0.27	1.93	2.54	1.90
X/9	2.39	2.54	2.55	2.58	2.58	2.58	+0.05	–0.01	+0.04	+0.00	2.66	–0.36	2.30	2.13	1.31

a All data are in kcal/mol.

b Conformer name based on the benchmark/MP2 relative energies.

c MP2/aug-cc-pVDZ relative energies at MP2/aug-cc-pVDZ geometries.

d CCSD(T)-F12a/cc-pVDZ-F12 relative energies at MP2/aug-cc-pVDZ geometries.

e CCSD(T)-F12a/cc-pVDZ-F12 relative energies at CCSD(T)-F12a/cc-pVDZ-F12 geometries.

f CCSD(T)-F12a/cc-pVTZ-F12 relative energies at CCSD(T)-F12a/cc-pVDZ-F12 geometries.

g CCSD(T)-F12a/cc-pVQZ-F12 relative energies at CCSD(T)-F12a/cc-pVDZ-F12 geometries.

h CCSD(T)-F12b/cc-pVQZ-F12 relative energies at CCSD(T)-F12a/cc-pVDZ-F12 geometries.

i Core-correlation correction obtained as the difference between AE-CCSD(T)-F12a/cc-pCVTZ-F12 and FC-CCSD(T)-F12a/cc-pCVTZ-F12 relative energies at CCSD(T)-F12a/cc-pVDZ-F12 geometries.

j Douglass–Kroll relativistic correction obtained as the difference between Douglas–Kroll AE-CCSD(T)/aug-cc-pwCVTZ-DK and AE-CCSD(T)/aug-cc-pwCVTZ relative energies at CCSD(T)-F12a/cc-pVDZ-F12 geometries.

k Full-T correction obtained by CCSDT – CCSD(T) with 6-31G basis set at CCSD(T)-F12a/cc-pVDZ-F12 geometries.

l Perturbative quadruples correction obtained by CCSDT(Q) – CCSDT with 6-31G basis set at CCSD(T)-F12a/cc-pVDZ-F12 geometries.

m Benchmark relative equilibrium energies obtained by CCSD(T)-F12b/cc-pVQZ-F12 + Δ_core_ + Δ_rel_ + δT + δ(Q).

n Zero-point corrections obtained by MP2/aug-cc-pVDZ harmonic vibrational frequencies.

o Benchmark adiabatic relative energies obtained as Δ*E*_e_ + Δ_ZPE_.

p Relative enthalpy at 298.15 K.

q Relative Gibbs free energy at 298.15 K.

The benchmark values for the N-, O-, and S-protonated cysteine can be seen in Tables 3, 4, and 5, respectively. The notation is similar to that for cysteine, but the subscript (in CCSD(T)-F12b/cc-pVQZ-F12 order) or the first character (MP2/aug-cc-pVDZ order) refers to the site of the protonation. The MP2/aug-cc-pVDZ geometries are accurate for the amino-protonated ones, the geometry effect is 0.02 kcal/mol on average, and for the carbonyl-protonated structures, the average grows larger to 0.06 kcal/mol. In the case of thiol protonation, we find that the geometry effect is much greater, 0.55 kcal/mol in general, while it is more than 1.00 kcal/mol for IX_S_! The differences between MP2/aug-cc-pVDZ and CCSD(T)-F12a/cc-pVDZ-F12 relative energies after the geometry optimization at the corresponding level of theory are 0.29 kcal/mol for N-, 0.21 kcal/mol for O-, and much higher, 1.15 kcal/mol for the S-protonated minima. Even in the energy order, we can observe many swaps between the MP2 and CCSD(T)-F12a results. In the case of the CCSD(T)-F12a method, the energy order of the conformers remains the same as we increase the basis set from cc-pVDZ-F12 to cc-pVQZ-F12. Speaking of that, here we can observe similar basis-set convergence as in the case of the neutral amino acid, except in the case of thiol protonation. The average difference between the CCSD(T)-F12a/cc-pVTZ-F12 and CCSD(T)-F12b/cc-pVQZ-F12 relative energies is still 0.04 kcal/mol, without outliers. The relative energy difference between the cc-pVQZ-F12 basis set used with the CCSD(T)-F12a and F12b methods again only 0.01 kcal/mol or less. The core correlation correction is always a positive value for all of the protonated species, 0.03 kcal/mol on average while the highest one is 0.07 kcal/mol for X_N_. The second-order Douglass–Kroll relativistic correction is always a positive value, except for IX_N_ and X_N_, and in general it is 0.01 kcal/mol; however, in a few cases (IV_S_, V_S_, VI_S_, VII_S_, VIII_S_) they can match the quantity of the core correction, as they are (0.03, 0.03, 0.04, 0.05, 0.03) kcal/mol, respectively. The sum of the post-(T) corrections is 0.03 kcal/mol on average with varying sign; in general, the δ(Q) component is much larger than δT. The sum of all of the corrections is 0.06 kcal/mol in general, but it can reach even 0.13 kcal/mol (for VI_S_ and VII_S_). With these, we obtain benchmark relative equilibrium energies, and unlike in the case of neutral cysteine, there is no change in energy order. Based on the MP2/aug-cc-pVDZ harmonic vibrational frequencies, we calculate the zero-point vibrational energy corrections. For the amino-protonated minima, the sign of the ZPE corrections varies, and the absolute corrections are usually around 0.11 kcal/mol. The carbonyl-protonated geometries have this correction with negative sign in every case, 0.47 kcal/mol on average. For the thiol-protonated conformers, the average ZPE correction becomes 0.79 kcal/mol, and it is a positive value for every one of them. Δ*E*_e_ + Δ_ZPE_ equals to benchmark adiabatic relative energies, and the order of the minima stays the same except for VI_S_ and VII_S_ as their order is switched. The thermal corrections leading to the 298.15 K enthalpy values are usually a few tenth kcal/mol, except for some outliers like VIII_S_, where it is over 1 kcal/mol. We finally obtain the 298.15 K Gibbs free energy values considering entropy effects. The amount of these effects varies between 0.01 and 1.34 kcal/mol, and it has negative sign except for VIII_S_. The uncertainty of the relative enthalpies and that of free energies are similar as in the case of cysteine.

**Table 3:** Benchmark Relative Energies, Corrections, Relative Enthalpies (at 0 and 298.15 K), and Relative Gibbs Free Energies (298.15 K) of the 10 Lowest-Lying N-Protonated Cysteine Conformers^a^

name^b^	MP2	CC//MP2	CCSD(T)-F12a			CCSD(T)-F12b	Δ_core_^i^	Δ_rel._^j^	δT^k^	δ(Q)^l^	Δ*E*_e_^m^	Δ_ZPE_^n^	Δ*H*_0_^o^	Δ*H*_298.15_^p^	Δ*G*_298.15_^q^
	aVDZ^c^	DZ^d^	DZ^e^	TZ^f^	QZ^g^	QZ^h^									
I_N_/N1	0.00	0.00	0.00	0.00	0.00	0.00	+0.00	+0.00	+0.00	+0.00	0.00	+0.00	0.00	0.00	0.00
II_N_/N2	0.52	0.52	0.54	0.56	0.55	0.55	+0.01	+0.00	+0.00	+0.00	0.56	+0.06	0.62	0.69	0.54
III_N_/N3	1.27	1.45	1.44	1.39	1.38	1.39	+0.00	+0.01	+0.00	+0.00	1.40	–0.14	1.27	1.37	1.04
IV_N_/N4	1.62	1.73	1.75	1.71	1.70	1.71	+0.01	+0.01	+0.00	+0.00	1.73	–0.06	1.67	1.83	1.36
V_N_/N5	2.77	3.16	3.19	3.20	3.20	3.20	+0.00	+0.00	–0.01	–0.01	3.18	+0.00	3.18	3.19	3.20
VI_N_/N6	4.07	4.40	4.38	4.43	4.42	4.43	+0.02	+0.00	–0.01	–0.02	4.41	–0.06	4.35	4.48	4.01
VII_N_/N7	4.46	4.94	4.96	4.93	4.93	4.93	+0.01	+0.00	–0.01	–0.01	4.90	–0.15	4.76	4.88	4.55
VIII_N_/N8	5.27	5.69	5.68	5.67	5.67	5.67	+0.02	+0.00	–0.01	–0.02	5.67	–0.18	5.49	5.72	4.96
IX_N_/N10	7.56	7.15	7.17	7.18	7.19	7.20	+0.06	–0.01	+0.01	+0.01	7.27	–0.14	7.14	7.32	6.73
X_N_/N9	7.54	7.31	7.31	7.31	7.32	7.33	+0.07	–0.01	+0.01	+0.00	7.39	–0.18	7.21	7.44	6.33

a All data are in kcal/mol.

b Conformer name based on the benchmark/MP2 relative energies.

c MP2/aug-cc-pVDZ relative energies at MP2/aug-cc-pVDZ geometries.

d CCSD(T)-F12a/cc-pVDZ-F12 relative energies at MP2/aug-cc-pVDZ geometries.

e CCSD(T)-F12a/cc-pVDZ-F12 relative energies at CCSD(T)-F12a/cc-pVDZ-F12 geometries.

f CCSD(T)-F12a/cc-pVTZ-F12 relative energies at CCSD(T)-F12a/cc-pVDZ-F12 geometries.

g CCSD(T)-F12a/cc-pVQZ-F12 relative energies at CCSD(T)-F12a/cc-pVDZ-F12 geometries.

h CCSD(T)-F12b/cc-pVQZ-F12 relative energies at CCSD(T)-F12a/cc-pVDZ-F12 geometries.

i Core-correlation correction obtained as the difference between AE-CCSD(T)-F12a/cc-pCVTZ-F12 and FC-CCSD(T)-F12a/cc-pCVTZ-F12 relative energies at CCSD(T)-F12a/cc-pVDZ-F12 geometries.

j Douglass–Kroll relativistic correction obtained as the difference between Douglas–Kroll AE-CCSD(T)/aug-cc-pwCVTZ-DK and AE-CCSD(T)/aug-cc-pwCVTZ relative energies at CCSD(T)-F12a/cc-pVDZ-F12 geometries.

k Full-T correction obtained by CCSDT – CCSD(T) with 6-31G basis set at CCSD(T)-F12a/cc-pVDZ-F12 geometries.

l Perturbative quadruples correction obtained by CCSDT(Q) – CCSDT with 6-31G basis set at CCSD(T)-F12a/cc-pVDZ-F12 geometries.

m Benchmark relative equilibrium energies obtained by CCSD(T)-F12b/cc-pVQZ-F12 + Δ_core_ + Δ_rel_ + δT + δ(Q).

n Zero-point corrections obtained by MP2/aug-cc-pVDZ harmonic vibrational frequencies.

o Benchmark adiabatic relative energies obtained as Δ*E*_e_ + Δ_ZPE_.

p Relative enthalpy at 298.15 K.

q Relative Gibbs free energy at 298.15 K.

**Table 4:** Benchmark Relative Energies, Corrections, Relative Enthalpies (at 0 and 298.15 K), and Relative Gibbs Free Energies (298.15 K) of the 10 Lowest-Lying O-Protonated Cysteine Conformers^a^

name^b^	MP2	CC//MP2	CCSD(T)-F12a			CCSD(T)-F12b	Δ_core_^i^	Δ_rel._^j^	δT^k^	δ(Q)^l^	Δ*E*_e_^m^	Δ_ZPE_^n^	Δ*H*_0_^o^	Δ*H*_298.15_^p^	Δ*G*_298.15_^q^
	aVDZ^c^	DZ^d^	DZ^e^	TZ^f^	QZ^g^	QZ^h^									
I_O_/O1	0.00	0.00	0.00	0.00	0.00	0.00	+0.00	+0.00	+0.00	+0.00	0.00	+0.00	0.00	0.00	0.00
II_O_/O2	1.00	1.12	1.12	1.07	1.07	1.07	+0.00	+0.01	+0.01	+0.00	1.09	–0.08	1.01	1.07	0.83
III_O_/O3	6.63	6.71	6.76	6.81	6.83	6.82	+0.04	+0.00	+0.01	+0.05	6.91	–0.70	6.21	6.24	5.91
IV_O_/O4	7.25	7.19	7.28	7.27	7.26	7.26	+0.04	+0.01	+0.00	+0.02	7.32	–0.35	6.97	7.05	6.80
V_O_/O6	7.84	7.46	7.56	7.57	7.56	7.55	+0.04	+0.01	–0.01	+0.06	7.65	–0.61	7.04	7.34	7.01
VI_O_/O7	7.93	7.55	7.65	7.66	7.66	7.65	+0.04	+0.00	+0.00	+0.07	7.76	–0.55	7.21	7.15	6.83
VII_O_/O5	7.62	7.70	7.78	7.78	7.79	7.79	+0.04	+0.00	+0.01	+0.05	7.88	–0.54	7.34	7.29	6.72
VIII_O_/O8	8.24	7.84	7.91	7.91	7.90	7.89	+0.05	+0.00	–0.01	+0.06	7.99	–0.51	7.48	7.58	7.13
IX_O_/O9	8.34	7.89	7.98	7.97	7.96	7.95	+0.02	+0.02	–0.01	+0.07	8.05	–0.41	7.63	7.81	7.31
X_O_/O10	8.43	8.51	8.60	8.54	8.53	8.53	+0.04	+0.02	+0.00	+0.02	8.60	–0.51	8.09	8.25	7.78

a All data are in kcal/mol.

b Conformer name based on the benchmark/MP2 relative energies.

c MP2/aug-cc-pVDZ relative energies at MP2/aug-cc-pVDZ geometries.

d CCSD(T)-F12a/cc-pVDZ-F12 relative energies at MP2/aug-cc-pVDZ geometries.

e CCSD(T)-F12a/cc-pVDZ-F12 relative energies at CCSD(T)-F12a/cc-pVDZ-F12 geometries.

f CCSD(T)-F12a/cc-pVTZ-F12 relative energies at CCSD(T)-F12a/cc-pVDZ-F12 geometries.

g CCSD(T)-F12a/cc-pVQZ-F12 relative energies at CCSD(T)-F12a/cc-pVDZ-F12 geometries.

h CCSD(T)-F12b/cc-pVQZ-F12 relative energies at CCSD(T)-F12a/cc-pVDZ-F12 geometries.

i Core-correlation correction obtained as the difference between AE-CCSD(T)-F12a/cc-pCVTZ-F12 and FC-CCSD(T)-F12a/cc-pCVTZ-F12 relative energies at CCSD(T)-F12a/cc-pVDZ-F12 geometries.

j Douglass–Kroll relativistic correction obtained as the difference between Douglas–Kroll AE-CCSD(T)/aug-cc-pwCVTZ-DK and AE-CCSD(T)/aug-cc-pwCVTZ relative energies at CCSD(T)-F12a/cc-pVDZ-F12 geometries.

k Full-T correction obtained by CCSDT – CCSD(T) with 6-31G basis set at CCSD(T)-F12a/cc-pVDZ-F12 geometries.

l Perturbative quadruples correction obtained by CCSDT(Q) – CCSDT with 6-31G basis set at CCSD(T)-F12a/cc-pVDZ-F12 geometries.

m Benchmark relative equilibrium energies obtained by CCSD(T)-F12b/cc-pVQZ-F12 + Δ_core_ + Δ_rel_ + δT + δ(Q).

n Zero-point corrections obtained by MP2/aug-cc-pVDZ harmonic vibrational frequencies.

o Benchmark adiabatic relative energies obtained as Δ*E*_e_ + Δ_ZPE_.

p Relative enthalpy at 298.15 K.

q Relative Gibbs free energy at 298.15 K.

**Table 5:** Benchmark Relative Energies, Corrections, Relative Enthalpies (at 0 and 298.15 K), and Relative Gibbs Free Energies (298.15 K) of the 10 Lowest-Lying S-Protonated Cysteine Conformers^a^

name^b^	MP2	CC//MP2	CCSD(T)-F12a			CCSD(T)-F12b	Δ_core_^i^	Δ_rel._^j^	δT^k^	δ(Q)^l^	Δ*E*_e_^m^	Δ_ZPE_^n^	Δ*H*_0_^o^	Δ*H*_298.15_^p^	Δ*G*_298.15_^q^
	aVDZ^c^	DZ^d^	DZ^e^	TZ^f^	QZ^g^	QZ^h^									
I_S_/S1	0.00	0.00	0.00	0.00	0.00	0.00	+0.00	+0.00	+0.00	+0.00	0.00	+0.00	0.00	0.00	0.00
II_S_/S3	1.97	0.66	0.81	0.83	0.79	0.79	+0.03	+0.01	–0.02	+0.06	0.86	+0.84	1.70	2.14	2.03
III_S_/S2	1.94	0.40	1.11	1.15	1.11	1.11	+0.02	+0.00	–0.01	+0.03	1.14	+0.87	2.01	1.81	1.76
IV_S_/S4	3.29	1.72	2.14	2.13	2.08	2.08	+0.03	+0.03	–0.03	+0.05	2.17	+0.61	2.78	3.11	1.77
V_S_/S5	3.44	2.17	2.49	2.48	2.44	2.43	+0.01	+0.03	–0.02	+0.02	2.48	+0.66	3.13	3.31	2.62
VI_S_/S6	4.44	2.66	3.06	3.03	2.98	2.98	+0.05	+0.04	–0.03	+0.07	3.11	+0.78	3.88	4.00	3.64
VII_S_/S7	4.52	2.71	3.12	3.07	3.03	3.02	+0.05	+0.05	–0.03	+0.07	3.16	+0.70	3.86	3.94	3.75
VIII_S_/S9	5.71	4.69	4.01	3.98	3.95	3.94	+0.04	+0.03	–0.06	+0.04	4.00	+1.18	5.18	6.19	6.69
IX_S_/S10	6.15	3.63	4.68	4.66	4.62	4.61	+0.06	+0.00	–0.04	+0.05	4.69	+0.78	5.47	4.99	4.51
X_S_/S8	5.32	4.26	5.03	5.07	5.04	5.04	+0.03	+0.00	–0.01	–0.03	5.03	+0.69	5.72	5.40	5.22

a All data are in kcal/mol.

b Conformer name based on the benchmark/MP2 relative energies.

c MP2/aug-cc-pVDZ relative energies at MP2/aug-cc-pVDZ geometries.

d CCSD(T)-F12a/cc-pVDZ-F12 relative energies at MP2/aug-cc-pVDZ geometries.

e CCSD(T)-F12a/cc-pVDZ-F12 relative energies at CCSD(T)-F12a/cc-pVDZ-F12 geometries.

f CCSD(T)-F12a/cc-pVTZ-F12 relative energies at CCSD(T)-F12a/cc-pVDZ-F12 geometries.

g CCSD(T)-F12a/cc-pVQZ-F12 relative energies at CCSD(T)-F12a/cc-pVDZ-F12 geometries.

h CCSD(T)-F12b/cc-pVQZ-F12 relative energies at CCSD(T)-F12a/cc-pVDZ-F12 geometries.

i Core-correlation correction obtained as the difference between AE-CCSD(T)-F12a/cc-pCVTZ-F12 and FC-CCSD(T)-F12a/cc-pCVTZ-F12 relative energies at CCSD(T)-F12a/cc-pVDZ-F12 geometries.

j Douglass–Kroll relativistic correction obtained as the difference between Douglas–Kroll AE-CCSD(T)/aug-cc-pwCVTZ-DK and AE-CCSD(T)/aug-cc-pwCVTZ relative energies at CCSD(T)-F12a/cc-pVDZ-F12 geometries.

k Full-T correction obtained by CCSDT – CCSD(T) with 6-31G basis set at CCSD(T)-F12a/cc-pVDZ-F12 geometries.

l Perturbative quadruples correction obtained by CCSDT(Q) – CCSDT with 6-31G basis set at CCSD(T)-F12a/cc-pVDZ-F12 geometries.

m Benchmark relative equilibrium energies obtained by CCSD(T)-F12b/cc-pVQZ-F12 + Δ_core_ + Δ_rel_ + δT + δ(Q).

n Zero-point corrections obtained by MP2/aug-cc-pVDZ harmonic vibrational frequencies.

o Benchmark adiabatic relative energies obtained as Δ*E*_e_ + Δ_ZPE_.

p Relative enthalpy at 298.15 K.

q Relative Gibbs free energy at 298.15 K.

### 3.4 Proton Affinity and Gas-Phase Basicity

The newly obtained proton affinity and gas-phase basicities with the auxiliary corrections can be seen in Table 6. We included both the 0 K and the 298.15 K values, since the latter ones can be used in practice. We also calculated these quantities for the protonation of the different sites, since if we use a global mixture, the amino protonation dominates the process, overshadowing the carbonyl and thiol protonation. The separation of the sites is even broken up further: there are values for the protonation of the global minimum to the global minimum of each site and the values for equilibrium mixtures. The population of the conformers in each case was calculated by Boltzmann-distribution, where needed. The effects of the previously computed corrections are also considered. The sign of the δT corrections is negative, except for the O protonation, and the δT values are in the 0.02–0.04 kcal/mol range, while the δ(Q) ones are also negative, except for the thiol protonation. The δ(Q) corrections are in the 0.01–0.04 kcal/mol range, and finally, the sum of the two corrections (post-(T) corrections) is negative. The post-(T) correction is the largest in case of the N-protonation (−0.04 kcal/mol considering only the cysteine conformer with the lowest energy and its lowest energy N-protonated counterpart, and −0.05 kcal/mol for the conformer mixtures), while it is −0.02 kcal/mol for every other site. The core correction is contributing more to the benchmark results, since it is 0.10 kcal/mol for the amino protonation, 0.05 or 0.04 kcal/mol for the carbonyl-protonation (if we consider the lowest energy conformers or mixtures, respectively), while it has the value of −0.06 kcal/mol for the thiol protonation. The last of these additions originates from the relativistic effect. It is a positive component in the range of 0.02–0.06 kcal/mol and is the most relevant for the S-protonation. The sum of these corrections is 0.09 kcal/mol for the amino, 0.05/0.04 kcal/mol (minima/mixture) for the carbonyl, and −0.03 kcal/mol for the thiol site. Based on the above correction values, we estimate an uncertainty of about 0.1 kcal/mol for the equilibrium PA values.

**Table 6:** Proton Affinity (at 0 and 298.15 K), Auxiliary Correction, and Gas-Phase Basicity (298.15 K) Values for the Different Sites of Cysteine (in kcal/mol)

	Δ*E*_QZ_^a^	δT^b^	δ(Q)^c^	Δ_core_^d^	Δ_rel._^e^	Δ*E*_e_^f^	Δ_ZPE_^g^	Δ*H*_0_^h^	Δ*H*_298.15_^i^	Δ*G*_298.15_^j^
I–I_N_	222.89	–0.03	–0.01	+0.10	+0.03	222.98	–8.49	214.49	215.79	208.44
I–I_O_	208.57	+0.02	–0.04	+0.05	+0.02	208.62	–7.45	201.17	202.74	194.56
I–I_S_	198.22	–0.03	+0.01	–0.06	+0.05	198.18	–5.46	192.72	194.02	186.86
average N^k^	223.60	–0.03	–0.01	+0.10	+0.04	223.69	–8.73	214.96	216.39	208.21
average O^k^	209.47	+0.02	–0.04	+0.04	+0.02	209.51	–7.67	201.83	203.55	194.16
average S^k^	199.13	–0.04	+0.02	–0.06	+0.06	199.10	–5.79	193.31	194.74	186.40

a CCSD(T)-F12b/cc-pVQZ-F12 equilibrium proton affinities at CCSD(T)-F12a/cc-pVDZ-F12 geometries.

b Full-T correction obtained by CCSDT – CCSD(T) with 6-31G basis set at CCSD(T)-F12a/cc-pVDZ-F12 geometries.

c Perturbative quadruples correction obtained by CCSDT(Q) – CCSDT with 6-31G basis set at CCSD(T)-F12a/cc-pVDZ-F12 geometries.

d Core-correlation correction obtained as the difference between AE-CCSD(T)-F12a/cc-pCVTZ-F12 and FC-CCSD(T)-F12a/cc-pCVTZ-F12 proton affinities at CCSD(T)-F12a/cc-pVDZ-F12 geometries.

e Douglass–Kroll relativistic correction obtained as the difference between Douglas–Kroll AE-CCSD(T)/aug-cc-pwCVTZ-DK and AE-CCSD(T)/aug-cc-pwCVTZ proton affinities at CCSD(T)-F12a/cc-pVDZ-F12 geometries.

f Benchmark equilibrium proton affinities obtained by CCSD(T)-F12b/cc-pVQZ-F12 + Δ_core_ + Δ_rel_ + δT + δ(Q).

g Zero-point corrections obtained by MP2/aug-cc-pVDZ harmonic vibrational frequencies.

h Benchmark 0 K proton affinities obtained as Δ*E*_e_ + Δ_ZPE_.

i Benchmark proton affinities (298.15 K).

j Benchmark gas-phase basicities (298.15 K).

k In the average mixtures, the population of the conformers were calculated by Boltzmann-distribution.

From the equilibrium PA values, we can get the enthalpy change of protonation at 0 K. The ZPE effect is roughly −8.5/–8.7; −7.5/–7.7; and −5.5/–5.8 kcal/mol depending on which protonation site we consider (minima/mixtures). The amino protonation Δ_ZPE_ value is lower, than in the case of glycine,^16^ but the carbonyl-protonation is almost the same (approximately −9.1 and −7.7 for both minima and mixtures). We convert the value to 298.15 K to get PAs by calculating rotational and vibrational thermal corrections and considering the translational enthalpy of the proton (1.48 kcal/mol at 298.15 K). This means an increase of 1.30/1.43 kcal/mol for the amino site, 1.57/1.72 kcal/mol for the carbonyl site, and finally, 1.30/1.43 kcal/mol for the thiol site. Finally, we get the gas-phase basicity values by calculating the entropy contributions. This lowers the PA values by 7.35/8.18; 8.17/9.39; and 7.16/8.34 kcal/mol. Both the PA and GB values differ greatly (practically 10–20 kcal/mol), depending on what protonation site we consider, the thermodynamically favored is the amino site, it is followed by the carbonyl site and finally the thiol one. Whereas the MP2/aug-cc-pVDZ frequencies cause considerable error in the individual thermodynamic values for the conformers, previously^16^ we found that for PA and GB values it is less serious. To approximate an uncertainty for the determined PA and GB values, we have to consider the following: the order of magnitude of the auxiliary corrections, the uncertainty of the low-frequency vibrations, and the neglected anharmonicity. The latter two are significant both in the thermal corrections and in the vibrational entropies. Finally, for the 298.15 K proton affinity of cysteine we suggest 216.39 ± 0.40 kcal/mol and for the 298.15 K gas-phase basicity, 208.21 ± 1.20 kcal/mol. The protonation of the thiol site can be useful for different applications, like solutions, where the amino acid is zwitterionic or when there are amino acid chains, but one should consider that not only solvation happens, but the chemical environment in the molecule also changes slightly.

There are theoretical publications in the literature already,^9,23,25,27,29^ but this work has many novelties, if one considers the number of conformers taken into account, the level of theory and corrections, and finally the consideration of the different protonation sites. From the experimental side, one can also find papers, which are not very recent. In 1992, Gorman^56^ obtained PA of 214.6 ± 2.7 kcal/mol from Fourier transform ion cyclotron resonance spectrometry experiments. Six years later, Hunter and Lias^57^ published an immense database of PA and GB for 1700 species. They collected and critically re-evaluated the state-of-the-art published data. For the cysteine at 298 K, they suggested 215.87 kcal/mol PA and 207.77 kcal/mol GB values, which are in good agreement with our theoretical predictions of 216.39 ± 0.40 and 208.21 ± 1.20 kcal/mol, respectively. Afonso et al.^58^ in 2000 performed electrospray ionization (ESI)-ion trap mass spectrometry measurements and obtained 214.41 kcal/mol for the PA of cysteine, although this value might not be considered as reliable as the former ones.^9^

## 4 Summary and Conclusions

We have mapped the conformational space of the neutral cysteine with six different basis sets, namely 3-21G, 6-31G, 6-31++G, 6-31G**, 6-31++G**, and cc-pVDZ using the MP2 method. The two latter bases proved to be the most efficient for the search of possible minimum geometries, combined together, as they both found unique conformers, but if computationally affordable, it might be useful to use even larger basis sets. Compared to the most adequate publication,^26^ we finally managed to locate 14 new minima, 85 in total at the MP2/aug-cc-pVDZ level of theory. The analysis of the occurrences in the data points showed that the search method is very sensitive to the starting geometry, and some conformers can be easily overlooked. We tested the possible protonation sites of cysteine, the thiol, amino, carbonyl, and the hydroxyl group. The protonated hydroxyl group is proven to be not stable. Based on our experience in the case of the neutral cysteine, we searched for the protonated cysteine conformers with the 6-31++G** and the cc-pVDZ basis sets combined with the MP2 method. We found 21 amino-, 64 carbonyl-, and 37 thiol-protonated structures, 122 in total, again at the MP2/aug-cc-pVDZ level of theory in comparison to the 21 reported in the literature.^53^

The 10 lowest-energy conformers of each group (the neutral cysteine and its N-, O- and S-protonated counterparts) were subjected for further benchmark investigations: from CCSD(T)-F12a/cc-pVDZ-F12 geometry optimization up to CCSD(T)-F12b/cc-pVQZ-F12 single-point energy computations. Core correlation correction, second-order Douglass–Kroll relativistic correction, and post-(T) correction (δT and δ(Q)) were also computed and shown to be necessary to achieve the desired sub-chemical accuracy. With the addition of these, we finally obtained the benchmark ab initio equilibrium relative energies for every conformer, with the Δ_ZPE_ we get the adiabatic relative energies. Applying statistical thermodynamics relative enthalpies and relative Gibbs free energies became available at 298.15 K.

Finally, these results of the cysteine and protonated cysteine yield us benchmark proton affinity and gas-phase basicity values. The protonation of the three different sites was considered, and the results showed that the protonation of the amino group is the most favored thermodynamically. We considered the protonation of the conformer with the lowest energy only and conformer mixtures too. For the PA, we calculated 216.39 ± 0.40 kcal/mol while for the gas-phase basicity 208.21 ± 1.20 kcal/mol. These are in good agreement with previous experimental work showing us, that like in the case of glycine,^16^ the relatively simple rigid rotator and harmonic oscillator models can lead to accurate absolute proton affinity values and gas-phase basicities. Hindered rotor or analytical frequency calculations could decrease the uncertainty of our thermodynamical values; nevertheless, the present results can still serve as benchmark references for new investigations.

## Supporting Information Available

MP2/aug-cc-pVDZ Cartesian coordinates (Å) and relative energies of the neutral cysteine conformers (XYZ)
MP2/aug-cc-pVDZ Cartesian coordinates (Å) and relative energies of “Broken” byproducts of the neutral cysteine conformer search (XYZ)
MP2/aug-cc-pVDZ Cartesian coordinates (Å) and relative energies of “Rearranged” byproducts of the neutral cysteine conformer search (XYZ)
MP2/aug-cc-pVDZ Cartesian coordinates (Å) and relative energies of N-protonated cysteine conformers (XYZ)
MP2/aug-cc-pVDZ Cartesian coordinates (Å) and relative energies of O-protonated cysteine conformers (XYZ)
MP2/aug-cc-pVDZ Cartesian coordinates (Å) and relative energies of S-protonated cysteine conformers (XYZ)
MP2/aug-cc-pVDZ Cartesian coordinates (Å) and relative energies of “Broken” byproducts of the protonated cysteine conformer search (XYZ)
MP2/aug-cc-pVDZ Cartesian coordinates (Å) and relative energies of “Cyclized” byproducts of the protonated cysteine conformer search (XYZ)
MP2/aug-cc-pVDZ Cartesian coordinates (Å) and relative energies of “Rearranged” byproducts of the protonated cysteine conformer search (XYZ)
CCSD(T)-F12a/cc-pVDZ-F12 Cartesian coordinates (Å) and relative energies of the 10 lowest-lying neutral cysteine conformers (XYZ)
CCSD(T)-F12a/cc-pVDZ-F12 Cartesian coordinates (Å) and relative energies of 10 lowest-lying N-protonated cysteine conformers (XYZ)
CCSD(T)-F12a/cc-pVDZ-F12 Cartesian coordinates (Å) and relative energies of 10 lowest-lying O-protonated cysteine conformers (XYZ)
CCSD(T)-F12a/cc-pVDZ-F12 Cartesian coordinates (Å) and relative energies of 10 lowest-lying S-protonated cysteine conformers (XYZ)

## Supplementary Material

jp2c07035_si_001.xyz

jp2c07035_si_002.xyz

jp2c07035_si_003.xyz

jp2c07035_si_004.xyz

jp2c07035_si_005.xyz

jp2c07035_si_006.xyz

jp2c07035_si_007.xyz

jp2c07035_si_008.xyz

jp2c07035_si_009.xyz

jp2c07035_si_010.xyz

jp2c07035_si_011.xyz

jp2c07035_si_012.xyz

jp2c07035_si_013.xyz

## References

[ref1] Sanz M. E.; Blanco S.; López J. C.; Alonso J. L. Rotational Probes of Six Conformers of Neutral Cysteine. Angew. Chem., Int. Ed. 2008, 47, 6216–6220. 10.1002/anie.200801337. 18618532

[ref2] Najbauer E. E.; Bazsó G.; Góbi S.; Magyarfalvi G.; Tarczay G. Exploring the Conformational Space of Cysteine by Matrix Isolation Spectroscopy Combined with Near-Infrared Laser Induced Conformational Change. J. Phys. Chem. B 2014, 118, 2093–2103. 10.1021/jp412550q. 24479484

[ref3] Ioppolo S.; Fedoseev G.; Chuang K.-J.; Cuppen H. M.; Clements A. R.; Jin M.; Garrod R. T.; Qasim D.; Kofman V.; van Dishoeck E. F.; Linnartz H. A Non-Energetic Mechanism for Glycine Formation in the Interstellar Medium. Nat. Astron. 2021, 5, 197–205. 10.1038/s41550-020-01249-0.

[ref4] Kuan Y.; Charnley S. B.; Huang H.; Tseng W.; Kisiel Z. Interstellar Glycine. Astrophys. J. 2003, 593, 848–867. 10.1086/375637.

[ref5] Snow J. L.; Orlova G.; Blagojevic V.; Bohme D. K. Gas-Phase Ionic Syntheses of Amino Acids: β versus α. J. Am. Chem. Soc. 2007, 129, 9910–9917. 10.1021/ja068725b. 17649994

[ref6] Herbst E. The Chemistry of Interstellar Space. Chem. Soc. Rev. 2001, 30, 168–176. 10.1039/a909040a.

[ref7] Blagojevic V.; Petrie S.; Bohme D. K. Gas-Phase Syntheses for Interstellar Carboxylic and Amino Acids. Mon. Not. R. Astron. Soc. 2003, 339, L7–L11. 10.1046/j.1365-8711.2003.06351.x.

[ref8] Maksić Z. B.; Kovačević B.; Vianello R. Advances in Determining the Absolute Proton Affinities of Neutral Organic Molecules in the Gas Phase and Their Interpretation: A Theoretical Account. Chem. Rev. 2012, 112, 5240–5270. 10.1021/cr100458v. 22857519

[ref9] Bleiholder C.; Suhai S.; Paizs B. Revising the Proton Affinity Scale of the Naturally Occurring α -Amino Acids. J. Am. Soc. Mass Spectrom. 2006, 17, 1275–1281. 10.1016/j.jasms.2006.05.010. 16829127

[ref10] Dongré A. R.; Jones J. L.; Somogyi Á.; Wysocki V. H. Influence of Peptide Composition, Gas-Phase Basicity, and Chemical Modification on Fragmentation Efficiency: Evidence for the Mobile Proton Model. J. Am. Chem. Soc. 1996, 118, 8365–8374. 10.1021/ja9542193.

[ref11] Moser A.; Range K.; York D. M. Accurate Proton Affinity and Gas-Phase Basicity Values for Molecules Important in Biocatalysis. J. Phys. Chem. B 2010, 114, 13911–13921. 10.1021/jp107450n. 20942500 PMC2970571

[ref12] Zhang K.; Chung-Phillips A. Gas-Phase Basicity of Glycine: A Comprehensive Ab Initio Study. J. Phys. Chem. A 1998, 102, 3625–3634. 10.1021/jp981405x.

[ref13] Zhang K.; Chung-Phillips A. A Computational Study of Intramolecular Proton Transfer in Gaseous Protonated Glycine. J. Chem. Inf. Comput. Sci. 1999, 39, 382–395. 10.1021/ci9802225. 10192949

[ref14] Topol I. A.; Burt S. K.; Toscano M.; Russo N. Protonation of Glycine and Alanine: Proton Affinities, Intrinsic Basicities and Proton Transfer Path. J. Mol. Struct.: THEOCHEM 1998, 430, 41–49. 10.1016/S0166-1280(98)90213-5.

[ref15] Orján E. M.; Nacsa A. B.; Czakó G. Conformers of Dehydrogenated Glycine Isomers. J. Comput. Chem. 2020, 41, 2001–2014. 10.1002/jcc.26375. 32579272

[ref16] Nacsa A. B.; Czakó G. Benchmark Ab Initio Proton Affinity of Glycine. Phys. Chem. Chem. Phys. 2021, 23, 9663–9671. 10.1039/D1CP00376C. 33908507

[ref17] Pepe C.; Rochut S.; Paumard J. P.; Tabet J. C. Ab Initio Calculations of Proton Affinities of Glycine, Proline, Cysteine and Phenylalanine: Comparison with the Experimental Values Obtained Using an Electrospray Ionisation Ion Trap Mass Spectrometer. Rapid Commun. Mass Spectrom. 2004, 18, 307–312. 10.1002/rcm.1330. 14755616

[ref18] Császár A. G. Conformers of Gaseous Glycine. J. Am. Chem. Soc. 1992, 114, 9568–9575. 10.1021/ja00050a041.

[ref19] Bazsó G.; Magyarfalvi G.; Tarczay G. Tunneling Lifetime of the Ttc/VIp Conformer of Glycine in Low-Temperature Matrices. J. Phys. Chem. A 2012, 116, 10539–10547. 10.1021/jp3076436. 23061476

[ref20] Conte R.; Houston P. L.; Qu C.; Li J.; Bowman J. M. Full-Dimensional, Ab Initio Potential Energy Surface for Glycine with Characterization of Stationary Points and Zero-Point Energy Calculations by Means of Diffusion Monte Carlo and Semiclassical Dynamics. J. Chem. Phys. 2020, 153, 244301 10.1063/5.0037175. 33380113

[ref21] Barone V.; Biczysko M.; Bloino J.; Puzzarini C. Characterization of the Elusive Conformers of Glycine from State-of-the-Art Structural, Thermodynamic, and Spectroscopic Computations: Theory Complements Experiment. J. Chem. Theory Comput. 2013, 9, 1533–1547. 10.1021/ct3010672. 26587615

[ref22] Gronert S.; O’Hair R. A. J. Ab Initio Studies of Amino Acid Conformations. 1. The Conformers of Alanine, Serine, and Cysteine. J. Am. Chem. Soc. 1995, 117, 2071–2081. 10.1021/ja00112a022.

[ref23] Maksić Z. B.; Kovačević B. Towards the Absolute Proton Affinities of 20 α-Amino Acids. Chem. Phys. Lett. 1999, 307, 497–504. 10.1016/S0009-2614(99)00535-7.

[ref24] Dobrowolski J. C.; Rode J. E.; Sadlej J. Cysteine Conformations Revisited. J. Mol. Struct.: THEOCHEM 2007, 810, 129–134. 10.1016/j.theochem.2007.02.011.

[ref25] Gronert S.; Simpson D. C.; Conner K. M. A Reevaluation of Computed Proton Affinities for the Common α-Amino Acids. J. Am. Soc. Mass Spectrom. 2009, 20, 2116–2123. 10.1016/j.jasms.2009.07.006. 19683940

[ref26] Wilke J. J.; Lind M. C.; Schaefer H. F.; Császár A. G.; Allen W. D. Conformers of Gaseous Cysteine. J. Chem. Theory Comput. 2009, 5, 1511–1523. 10.1021/ct900005c. 26609845

[ref27] Brás N. F.; Perez M. A. S.; Fernandes P. A.; Silva P. J.; Ramos M. J. Accuracy of Density Functionals in the Prediction of Electronic Proton Affinities of Amino Acid Side Chains. J. Chem. Theory Comput. 2011, 7, 3898–3908. 10.1021/ct200309v. 26598336

[ref28] Hernández B.; Pflüger F.; Adenier A.; Kruglik S. G.; Ghomi M. Side Chain Flexibility and Protonation States of Sulfur Atom Containing Amino Acids. Phys. Chem. Chem. Phys. 2011, 13, 17284 10.1039/c1cp21054h. 21879053

[ref29] Riffet V.; Frison G.; Bouchoux G. Acid–Base Thermochemistry of Gaseous Oxygen and Sulfur Substituted Amino Acids (Ser, Thr, Cys, Met). Phys. Chem. Chem. Phys. 2011, 13, 18561 10.1039/c1cp22206f. 21947236

[ref30] Freeman F.; Adesina I. T.; Le La J.; Lee J. Y.; Poplawski A. A. Conformers of Cysteine and Cysteine Sulfenic Acid and Mechanisms of the Reaction of Cysteine Sulfenic Acid with 5,5-Dimethyl-1,3-Cyclohexanedione (Dimedone). J. Phys. Chem. B 2013, 117, 16000–16012. 10.1021/jp409022m. 24274619

[ref31] Dinadayalane T. C.; Sastry G. N.; Leszczynski J. Comprehensive Theoretical Study towards the Accurate Proton Affinity Values of Naturally Occurring Amino Acids. Int. J. Quantum Chem. 2006, 106, 2920–2933. 10.1002/qua.21117.

[ref32] Mancini G.; Fusè M.; Lazzari F.; Chandramouli B.; Barone V. Unsupervised Search of Low-Lying Conformers with Spectroscopic Accuracy: A Two-Step Algorithm Rooted into the Island Model Evolutionary Algorithm. J. Chem. Phys. 2020, 153, 124110 10.1063/5.0018314. 33003701

[ref33] Ferro-Costas D.; Mosquera-Lois I.; Fernández-Ramos A. TorsiFlex: An Automatic Generator of Torsional Conformers. Application to the Twenty Proteinogenic Amino Acids. J. Cheminform. 2021, 13, 100 10.1186/s13321-021-00578-0. 34952644 PMC8710030

[ref34] Ropo M.; Schneider M.; Baldauf C.; Blum V. First-Principles Data Set of 45,892 Isolated and Cation-Coordinated Conformers of 20 Proteinogenic Amino Acids. Sci. Data 2016, 3, 160009 10.1038/sdata.2016.9. 26881946 PMC4755128

[ref35] Kishimoto N.; Waizumi H. An Automated and Efficient Conformation Search of L-Cysteine and L,L-Cystine Using the Scaled Hypersphere Search Method. Chem. Phys. Lett. 2017, 685, 69–76. 10.1016/j.cplett.2017.07.029.

[ref36] Dékány A. Á.; Czakó G. Benchmark Ab Initio Proton Affinity and Gas-phase Basicity of α-Alanine Based on Coupled-cluster Theory and Statistical Mechanics. J. Comput. Chem. 2022, 43, 19–28. 10.1002/jcc.26767. 34676890

[ref37] Hehre W. J.; Ditchfield R.; Pople J. A. Self—Consistent Molecular Orbital Methods. XII. Further Extensions of Gaussian—Type Basis Sets for Use in Molecular Orbital Studies of Organic Molecules. J. Chem. Phys. 1972, 56, 2257–2261. 10.1063/1.1677527.

[ref38] Dunning T. H. Gaussian Basis Sets for Use in Correlated Molecular Calculations. I. The Atoms Boron through Neon and Hydrogen. J. Chem. Phys. 1989, 90, 1007–1023. 10.1063/1.456153.

[ref39] Møller C.; Plesset M. S. Note on an Approximation Treatment for Many-Electron Systems. Phys. Rev. 1934, 46, 618–622. 10.1103/PhysRev.46.618.

[ref40] Werner H.-J.; Knowles P. J.; Knizia G.; Manby F. R.; Schütz M. MOLPRO, version 2015.1, a package of ab initio programs. www.molpro.net (accessed November 2022).

[ref41] Kállay M.; Nagy P. R.; Mester D.; Gyevi-Nagy L.; Csóka J.; Szabó P. B.; Rolik Z.; Samu G.; Csontos J.; Hégely B.; MRCC, a quantum chemical program suite. www.mrcc.hu (accessed November 2022).

[ref42] Kállay M.; Nagy P. R.; Mester D.; Rolik Z.; Samu G.; Csontos J.; Csóka J.; Szabó P. B.; Gyevi-Nagy L.; Hégely B.; et al. The MRCC Program System: Accurate Quantum Chemistry from Water to Proteins. J. Chem. Phys. 2020, 152, 074107 10.1063/1.5142048. 32087669

[ref43] Adler T. B.; Knizia G.; Werner H.-J. A Simple and Efficient CCSD(T)-F12 Approximation. J. Chem. Phys. 2007, 127, 221106 10.1063/1.2817618. 18081383

[ref44] Knizia G.; Adler T. B.; Werner H.-J. Simplified CCSD(T)-F12 Methods: Theory and Benchmarks. J. Chem. Phys. 2009, 130, 054104 10.1063/1.3054300. 19206955

[ref45] Peterson K. A.; Adler T. B.; Werner H.-J. Systematically Convergent Basis Sets for Explicitly Correlated Wavefunctions: The Atoms H, He, B–Ne, and Al–Ar. J. Chem. Phys. 2008, 128, 084102 10.1063/1.2831537. 18315028

[ref46] Noga J.; Bartlett R. J. The Full CCSDT Model for Molecular Electronic Structure. J. Chem. Phys. 1987, 86, 7041–7050. 10.1063/1.452353.

[ref47] Kállay M.; Gauss J. Approximate Treatment of Higher Excitations in Coupled-Cluster Theory. J. Chem. Phys. 2005, 123, 214105 10.1063/1.2121589. 16356037

[ref48] Hill J. G.; Mazumder S.; Peterson K. A. Correlation Consistent Basis Sets for Molecular Core-Valence Effects with Explicitly Correlated Wave Functions: The Atoms B–Ne and Al–Ar. J. Chem. Phys. 2010, 132, 054108 10.1063/1.3308483. 20136306

[ref49] Douglas M.; Kroll N. M. Quantum Electrodynamical Corrections to the Fine Structure of Helium. Ann. Phys. 1974, 82, 89–155. 10.1016/0003-4916(74)90333-9.

[ref50] Raghavachari K.; Trucks G. W.; Pople J. A.; Head-Gordon M. A Fifth-Order Perturbation Comparison of Electron Correlation Theories. Chem. Phys. Lett. 1989, 157, 479–483. 10.1016/S0009-2614(89)87395-6.

[ref51] de Jong W. A.; Harrison R. J.; Dixon D. A. Parallel Douglas–Kroll Energy and Gradients in NWChem: Estimating Scalar Relativistic Effects Using Douglas–Kroll Contracted Basis Sets. J. Chem. Phys. 2001, 114, 48 10.1063/1.1329891.

[ref52] Pecul M. Conformational Structures and Optical Rotation of Serine and Cysteine. Chem. Phys. Lett. 2006, 418, 1–10. 10.1016/j.cplett.2005.09.137.

[ref53] Noguera M.; Rodríguez-Santiago L.; Sodupe M.; Bertran J. Protonation of Glycine, Serine and Cysteine. Conformations, Proton Affinities and Intrinsic Basicities. J. Mol. Struct.: THEOCHEM 2001, 537, 307–318. 10.1016/S0166-1280(00)00686-2.

[ref54] Szidarovszky T.; Czakó G.; Császár A. G. Conformers of Gaseous Threonine. Mol. Phys. 2009, 107, 761–775. 10.1080/00268970802616350.

[ref55] Barone V.; Biczysko M.; Bloino J.; Puzzarini C. Accurate Structure, Thermodynamic and Spectroscopic Parameters from CC and CC/DFT Schemes: The Challenge of the Conformational Equilibrium in Glycine. Phys. Chem. Chem. Phys. 2013, 15, 10094–10111. 10.1039/c3cp50439e. 23599122 PMC4596005

[ref56] Gorman G. S.; Speir J. P.; Turner C. A.; Amster I. J. Proton Affinities of the 20 Common α-Amino Acids. J. Am. Chem. Soc. 1992, 114, 3986–3988. 10.1021/ja00036a062.

[ref57] Hunter E. P. L.; Lias S. G. Evaluated Gas Phase Basicities and Proton Affinities of Molecules: An Update. J. Phys. Chem. Ref. Data 1998, 27, 413–656. 10.1063/1.556018.

[ref58] Afonso C.; Modeste F.; Breton P.; Fournier F.; Tabet J.-C. Proton Affinities of the Commonly Occuring L-Amino Acids by Using Electrospray Ionization-Ion Trap Mass Spectrometry. Eur. J. Mass Spectrom. 2000, 6, 443–449. 10.1255/ejms.369.

